# TGF-β Prevents Phosphate-Induced Osteogenesis through Inhibition of BMP and Wnt/β-Catenin Pathways

**DOI:** 10.1371/journal.pone.0089179

**Published:** 2014-02-27

**Authors:** Fátima Guerrero, Carmen Herencia, Yolanda Almadén, Julio M. Martínez-Moreno, Addy Montes de Oca, María Encarnación Rodriguez-Ortiz, Juan M. Diaz-Tocados, Antonio Canalejo, Mónica Florio, Ignacio López, William G. Richards, Mariano Rodriguez, Escolástico Aguilera-Tejero, Juan R. Muñoz-Castañeda

**Affiliations:** 1 Instituto Maimónides para la Investigación Biomédica (IMIBIC). Hospital Reina Sofía. Universidad de Córdoba, Córdoba, Spain; 2 Lipid and Atherosclerosis Unit. IMIBIC/Reina Sofía University Hospital/University of Córdoba, and CIBER Fisiopatología Obesidad y Nutrición (CIBEROBN), Instituto de Salud Carlos III, Córdoba, Spain; 3 Departamento de Biología Ambiental y Salud Pública, Universidad de Huelva, Huelva, Spain; 4 Metabolic Disorders Department, Amgen, Inc. One Amgen Center Drive, Thousand Oaks, California, United States of America; 5 Department of Medicina y Cirugía Animal, Universidad de Cordoba, Cordoba, Spain; National Institute of Health and Medical Research, France

## Abstract

**Background:**

Transforming growth factor-β (TGF-β) is a key cytokine during differentiation of mesenchymal stem cells (MSC) into vascular smooth muscle cells (VSMC). High phosphate induces a phenotypic transformation of vascular smooth muscle cells (VSMC) into osteogenic-like cells. This study was aimed to evaluate signaling pathways involved during VSMC differentiation of MSC in presence or not of high phosphate.

**Results:**

Our results showed that TGF-β induced nuclear translocation of Smad3 as well as the expression of vascular smooth muscle markers, such as smooth muscle alpha actin, SM22α, myocardin, and smooth muscle-myosin heavy chain. The addition of high phosphate to MSC promoted nuclear translocation of Smad1/5/8 and the activation of canonical Wnt/β-catenin in addition to an increase in BMP-2 expression, calcium deposition and alkaline phosphatase activity. The administration of TGF-β to MSC treated with high phosphate abolished all these effects by inhibiting canonical Wnt, BMP and TGF-β pathways. A similar outcome was observed in high phosphate-treated cells after the inhibition of canonical Wnt signaling with Dkk-1. Conversely, addition of both Wnt/β-catenin activators CHIR98014 and lithium chloride enhanced the effect of high phosphate on BMP-2, calcium deposition and alkaline phosphatase activity.

**Conclusions:**

Full VSMC differentiation induced by TGF-β may not be achieved when extracellular phosphate levels are high. Moreover, TGF-β prevents high phosphate-induced osteogenesis by decreasing the nuclear translocation of Smad 1/5/8 and avoiding the activation of Wnt/β-catenin pathway.

## Introduction

Vascular calcification (VC) is a process frequently observed in the elderly and in patients with diabetes or chronic kidney disease (CKD). Several clinical studies have demonstrated that VC is independently associated with increased cardiovascular morbidity and mortality [1], [2]. The calcification of the vascular wall, particularly medial calcification, is an organized process that involves phenotypic transformation of VSMC into an osteogenic cell type capable of secreting those proteins required for calcification [3]–[5]. Experimental works have demonstrated that an increase in extracellular phosphate, which is frequently observed in uremic patients, causes osteogenic transdifferentiation of VSMC [4], [6], [7].

Progenitors of VSMC are originated from the multipotent mesenchymal stem cells (MSC) present in the bone-marrow. These MSC may differentiate into VSMC in the presence of TGF- β [8], [9]. As high phosphate promotes osteogenesis in the vascular wall we thought that it is important to learn whether high phosphate has any effect on TGF-β induced VSMC differentiation. Therefore, this study was aimed to evaluate the signaling pathways involved in the regulation of the differentiation toward VSMC and osteogenesis induced by TGF and high phosphate in cultured MSC.

Our results show a crosstalk between the effects triggered by TGF-β and high phosphate during the differentiation of MSC into VSMC. Phosphate hinders VSMC differentiation induced by TGF-β while TGF-β prevents osteogenesis induced by high phosphate.

## Materials and Methods

### Ethics Statement

All experimental protocols were reviewed and approved by the Ethics Committee for Animal Research of the University of Cordoba in accordance with the ethical guidelines of the Institution and the EC Directive 86/609/EEC for animal experiments. Ten male Wistar rats were euthanized by aortic puncture and exsanguination under general anesthesia with pentobarbital sodium (50 mg/kg) and midazolam (4 mg/kg i.p).

### Rat Mesenchymal Stem Cells (MSC) isolation

Tibias and femurs of rats were cut at the epiphyses and perfused with alpha minimal essential medium (αMEM) medium (Sigma-Aldrich, St. Louis, MO) supplemented with 15% fetal bovine serum (FBS) (Lonza Walkersville, Inc., USA). After centrifugation and washing with αMEM medium, bone marrow stem cells were filtered and plated in 25 cm^2^ flasks (Corning Life Sciences – ALP, Chorges, France) with αMEM + 15% FBS and 1 ng/mL of basic fibroblast growth factor (bFGF) (PeproTech EC Ltd, London, UK). Fresh αMEM medium with 10% FBS and bFGF was added after 48h and successively changed 2 to 3 times per week. After reaching 85% to 90% of confluence, cells were collected using Trypsin-EDTA (Lonza Walkersville, Inc., USA) and seeded in 6-well plates at 13000 cells/cm^2^ in αMEM + bFGF. Treatments were started as described below when cells reached confluence. Experiments were performed three times.

### Treatments

VSMC differentiation was induced by treating MSC with 1 ng/mL of Transforming Growth Factor-β (TGF-β) (PeproTech EC Ltd, London, UK) for 14 days. Differentiated (+TGF-β) and undifferentiated (-TGF-β) MSC were cultured in the presence or absence of 10 mM β-glycerophosphate (P) (4,2 mM of phosphate) (Sigma-Aldrich, St. Louis, MO) to simulate a high phosphate environment. Undifferentiated (-TGF-β) MSC were treated either with an inhibitor of the Wnt/β-catenin pathway, recombinant dickkopf-related protein 1 (Dkk-1) (30 ng/mL) (R&D Systems, Inc, Minneapolis, MN), or with activators of the Wnt/β-catenin pathway, CHIR98014 (0.4 µM) [10] (donated by Amgen Inc., Thousand Oaks, CA) or lithium chloride (LiCl) (5 mM) (Sigma-Aldrich). Wnt/β-catenin activation/inhibition was assessed by confocal immunostaining and RT-PCR, as described below. Recombinant noggin (R&D Systems, MN, USA) was administered to high phosphate treated cells at a dose of 200 ng/ml.

### Gene expression analysis

Total RNA was extracted with Tri-Reagent™ (Sigma-Aldrich) and quantified by spectrophotometry (ND-1000, Nanodrop Technologies, Wilmington, DE). Vascular smooth muscle alpha-actin (VSM-actin), smooth muscle protein 22 alpha (SM22α), myocardin (Myocd), smooth muscle*-*myosin heavy chain (Myhc), Runt-related transcription factor 2 (Runx2), osterix (Sp7 transcription factor), bone morphogenetic protein 2 (BMP2), Dickkopf (Dkk1), low density lipoprotein receptor-related protein 5 (Lrp5) and glycogen synthase kinase 3 beta (Gsk3β) mRNA levels were determined by quantitative real-time RT-PCR (Light cycler, Roche Diagnostics, Basel, Switzerland). cDNA was synthesized from 0.5 µg of total RNA with a first strand cDNA synthesis kit (Qiagen, Hilden, Germany) in the presence of random hexamers in a final volume of 20 µl followed by 42°C for 15 min and 95°C for 3 min. An RT-PCR SYBR Green kit (Qiagen) was used to quantify mRNA expression levels. The primers used for PCR are shown in Table 1. mRNA expression was expressed as a value normalized to levels of 18S RNA.

**Table 1:** Primer sequences used for RT-PCR.

Gene Symbol	Gene Name	Forward	Reverse
Myhc	Smooth muscle-Myosin heavy chain	5′-GAGAATGAGAAGAAAGCCAAGAG-3′	5′-CATCCAGCTCCCGCTGCAGCT-3′
VSM-actin	Smooth muscle alpha-actin	5′- GACACCAGGGAGTGATGGTT-3′	5′- GTTAGCAAGGTCGGATGCTC-3′
Myocd	Myocardin	5'-CTCGGAGTCAGCAGATGGATG-3′	5′-CCTCACTGTCGGTGGCATAGT-3′
SM22	Smooth muscle protein 22 alpha (SM22α, transgelin).	5′- CAC CTA TCC TCA GCC TCA GC-3′	5′- TCC AAA GGA CAT TGG CTT CC-3′
Osterix	Osterix; Sp7 transcription factor	5'- GTACGGCAAGGCTTCGCATCTGA- 3'	5′-TCAAGTGGTCGCTTCGGGTAAAG -3′
Runx2	Runt-related transcription factor 2	5'- CGG GAATGATGAGAACTACTC-3'	5'- GCG GTCAGAGAACAAACTAGG T -3'
BMP-2	Bone morphogenetic protein 2	5'-AAGGCTTCTTCTTGCTGGTG-3'	5'-GCCTTACCCTCATGATGTCC-3'
Dkk1	Dickkopf 1	5' ACA ACT ACC AGC CCT ACC CTT 3'	5' CCT TCT TGC CCT TTG GTG TGA TA 3'
Lrp5	Low density lipoprotein receptor-related protein 5	5′-TGTGCCACTGGTGAG ATTGACT -3′	5′- ACGCTGGCAGACAAAGTAGAC -3′
Gsk3β	Glycogen synthase kinase 3 beta	5'-AGC ATG AAA GTT AGC AGA GAC 3'	5' TCG ATT CTT AAA TCT CTT GTC C 3'
18S rRNA	18S ribosomal RNA	5′-GTAACCCGTTGAACCCCATT-3′	5′- CCATCCAATCGGTAGTAGCG-3′

### Protein extracts

Cytosolic protein was isolated from MSC in a lysis buffer containing 10 mM Hepes, 10 mM KCl, 0.1 mM EDTA, 0.1 mM EGTA, 1 mM DTT, 0.5 mM PMSF, 70 µg/mL Protease Inhibitor Cocktail, 0.5% Igepal CA-630, pH 7.9. The suspensions were centrifuged and the supernatants (cytosolic extracts) were stored. Nuclear extracts were obtained by incubating the pellets obtained from the cytosolic extract in a lysis buffer containing 20 mM HEPES, 0.4 mM NaCl, 1 mM EDTA, 1 mM EGTA, 1 mM DTT, 1 mM PMSF, 46 µg/mL Protease Inhibitor Cocktail, pH 7.9. Protein concentration was determined by the Bradford assay (Bio-Rad Laboratories GmbH, Munich, Germany).

### Nucleofection of TCF/LEF reporter and dual-luciferase reporter assays

Rat MSC (4×10^5^) in log-phase growth were electroporated using the program U23 of the Amaxa Human MSC nucleofector kit (Lonza Cologne GmbH, Germany). Cells were transfected with 2 µg of pGL3-OT (Addgene, Cambridge, MA) and 100 ng of pRL-CMV (Promega), which expresses Renilla luciferase and was used to normalize the transfection efficiency according to the manufacturer’s instructions. After nucleofection, cells were cultured at 40000 cell/cm^2^ in 96-well plates with Mesenchymal Stem Cell Growth Medium BulletKit (Clonetics/BioWhittaker, Lonza) for 24 h. High phosphate (10 mM, BGP) was added and cells were lysed after 24 h of treatment. Luciferase activity was measured using the Dual Glo-Luciferase Reporter Assay System (Promega, WI, USA) and a luminometer (TECAN infinite F200 Pro) according to the manufacturer's protocol. Three independent experiments were performed.

### Confocal Immunostaining

Cells were fixed with cold methanol for 20 min and subsequently washed with PBS. Fixed cells were incubated for 1 h with primary antibodies against actin-alpha-2 (protein product of the *Acta2* gene; Genetex Inc, CA, USA), phospho-Smad3 (Ser 423/425, clone C25A9) and phospho-Smad1 (Ser 463/465)/Smad 5 (Ser 463/465)/Smad 8 (Ser 426/428) (phospho-Smad 1/5/8) (Cell Signaling Technology, MA, USA) and β-catenin (BD Biosciences, CA, USA). Cells were then incubated for 1 h at room temperature with Alexa Fluor 488 or Alexa Fluor 568 anti-mouse secondary antibodies (Invitrogen Ltd, Paisley, UK). Cell nuclei were visualized with the nuclear stain 4′, 6-diamino-2-phenylindole dihydrochloride (DAPI; Invitrogen Ltd, Paisley, UK). Pictures were obtained at 40x in an Axio Observer.Z1 Inverted Confocal microscope (LSM5 Exciter Zeiss). ImageJ software was used to analyze confocal images.

### Alkaline Phosphatase Activity

2 µg of protein were used to colorimetrically (OD 405nm) measure alkaline phosphatase specific activity with 5 mM p-nitrophenolphosphate (Sigma-Aldrich) used as a substrate. Cell lysates were incubated in 2 mM p-nitrophenol phosphate for 30 min at 37°C. The reaction was stopped by adding 1 M NaOH, and the product was quantified at 405 nm. One unit of alkaline phosphatase was defined as 1 µmol substrate hydrolyzed per hour (perµg protein/sample).

### Calcium content of cells

Cells were decalcified with 0.6 N HCl for 24 h at 37°C, and the calcium content in the HCl supernatant was determined by the o-cresolphthaleincomplexone method (Calcium C-Test, WAKO GmbH, Neuss, Germany). Cells were washed 3 times with PBS and solubilized in 0.1 mol/L NaOH 0.1% sodium dodecyl sulfate (SDS). Cell protein content was measured by the Bradford assay. Calcium content was normalized to total protein.

### Statistical Analysis

Values are shown as mean ± SE. Differences between groups were compared using ANOVA followed by a *post hoc* analysis (Duncan) or pair-wise *t* tests. P-value < 0.05 was considered statistically significant. Analyses were conducted using SPSS software (version 15.0, Somers, NY).

## Results

### Immunophenotype analysis of isolated bone marrow stem cells

Stem cells obtained from bone marrow exhibited the surface proteins CD90, CD105, CD29 and Sca-1, which characterize the immunophenotype of MSC (Figure S1A, Supporting information). The multipotent potential of MSC to differentiate into other cell types such as osteoblasts (Figure S1B, Supporting information) or adipocytes (Figure S1C, Supporting information) was also demonstrated.

### TGF-β induced vascular smooth muscle cell differentiation includes nuclear translocation of phospho-Smad3

**Figure 1:**
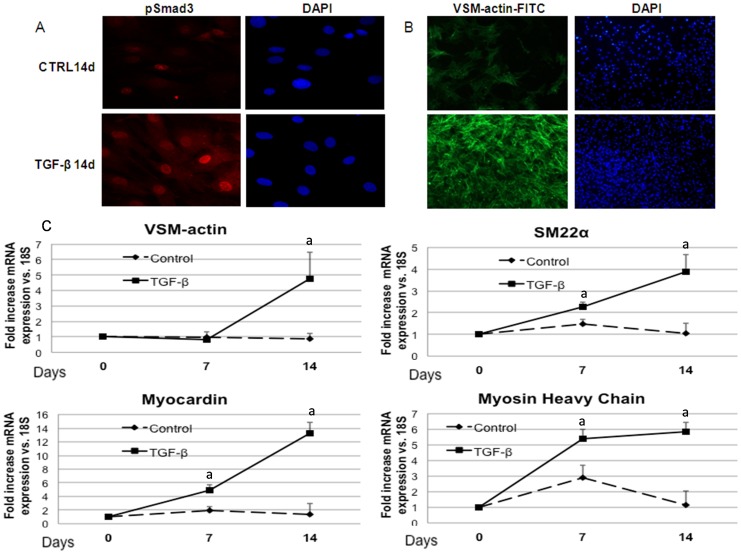
TGF-β induces vascular smooth muscle cells differentiation of mesenchymal stem cells through nuclear translocation of Smad3. A) Rat mesenchymal cells treated with TGF-β for 14 days were stained for phospho-Smad3 immunofluorescence (red) and counterstained with DAPI (blue) to determine phospho-Smad3 subcellular localization. In TGF-β treated cells, positive phospho-Smad3 immunofluorescence was localized into the nucleus. Original magnification: 40x. B) Vascular smooth muscle actin (VSM-actin, green) was stained and the nuclei were counter-stained with DAPI showing cytoskeleton organization in Control cells and TGF-β treated cells. Original magnification: 20x.C) After 7 and 14 days, TGF-β induced the expression of vascular smooth muscle cells markers such as VSM-actin, SM22α, Myocardin and Myosin heavy chain with respect to control cells (a p<0.001 vs. control cells). Images are representative of three experiments.

In cultured MSC, the addition of TGF-β produced nuclear translocation of phospho-Smad3 (Figure 1A). The presence of TGF-β was associated with the expression of proteins that characterize the VSMC phenotype. VSM-actin protein was significantly increased with respect to undifferentiated MSC (Figure 1B). Genes such as SM22α, myocardin or myosin heavy chain were up-regulated after 7 and 14 days of culture with TGF-β, while VSM-actin was significantly up-regulated only after 14 days (Figure 1C).

### Effect of high phosphate on vascular smooth muscle cell differentiation induced by TGF-β

**Figure 2:**
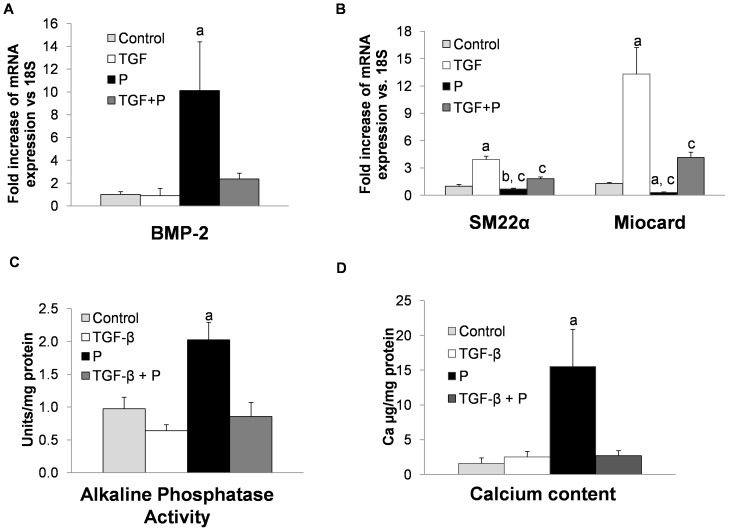
TGF-β administration prevents osteogenic effects induced by high phosphate. A) High phosphate (P) increased the expression of BMP-2 while TGF-β or the combination of TGF-β plus high phosphate decreased significantly the expression of this osteogenic marker (a p<0.001 vs. all groups). Results are expressed as fold change vs. Control cells. B) High phosphate (P) decreased significantly SM22α and myocardin expression with respect to Control cells (b p<0.01 for SM22α and a p<0.001 for myocardin) and TGF-β group (c p<0.001). The combination of TGF- β and high phosphate (TGF-β + P) decreased the expression of SM22α and Myocardin although less than high phosphate alone (c p <0.001vs. TGF-β group). C) TGF-β alone did not change significantly the alkaline phosphatase activity. This activity increased after high phosphate treatment (a p<0.001 vs. all others groups). The combination of TGF-β and high phosphate for 14 days significantly decreased this activity when compared with high phosphate group. D) Calcium content was significantly increased after high phosphate treatment (a p<0.001 vs other groups). The combination of TGF-β and high phosphate prevented this increase of calcium induced by high phosphate alone.

As shown in Figure 2A, addition of high phosphate alone to MSC cultures resulted in a high expression of BMP-2, a key factor that promotes osteogenic differentiation. However when MSC were cultured with TGF-β and high phosphate, the expression of BMP-2 was markedly reduced. In addition, the expression of VSMC specific markers (SM22α and myocardin) increased with respect to cells incubated only with high phosphate, although it was significantly lower than in the TGF-β-treated cells (Figure 2B). Furthermore, the presence of TGF-β prevented the increase in alkaline phosphatase activity and calcium deposition induced by high phosphate (Figure 2C and 2D).

**Figure 3:**
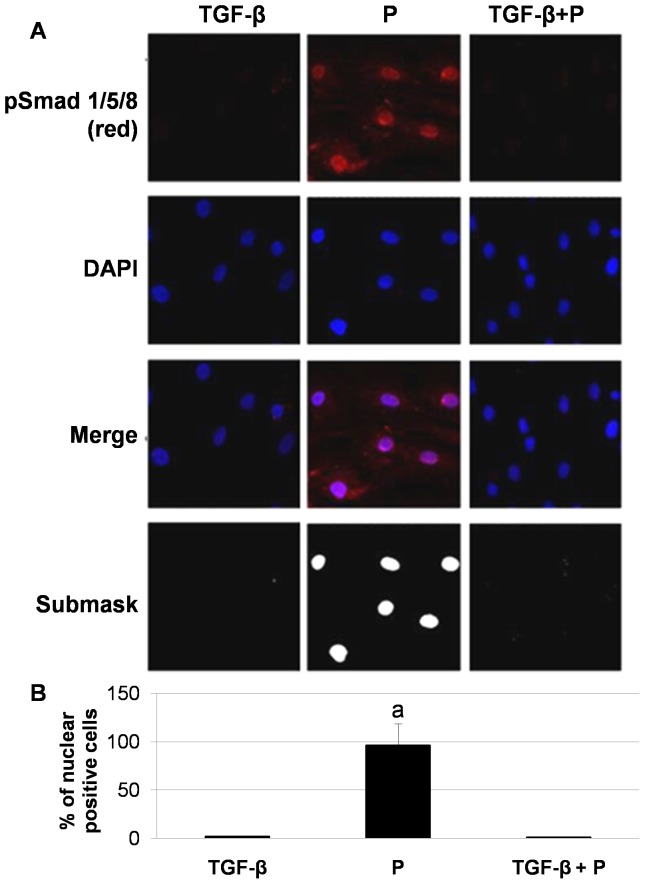
TGF-β addition inhibits nuclear translocation of Smad 1/5/8 induced by high Phosphate. A) Rat mesenchymal stem cells treated with high phosphate showed nuclear localization of phospho-Smad1/5/8 (Red) (a p<0.001 vs. all groups). Cells treated with TGF-β (alone or plus high phosphate) were negative for phospho-Smad1/5/8. Merged images of phospho-Smad1/5/8 immunofluorescence and DAPI staining are shown. Original magnification: 40x. Image is representative of three experiments. Colocalization Finder plugging from Image J software was carried out to analyse nuclear localization of Smad 1/5/8 showing a submask with white areas specific to nuclear colocalization with DAPI. Original magnification: 40x. B) Quantification of confocal immunofluorescence was performed with Image J software.

**Figure 4:**
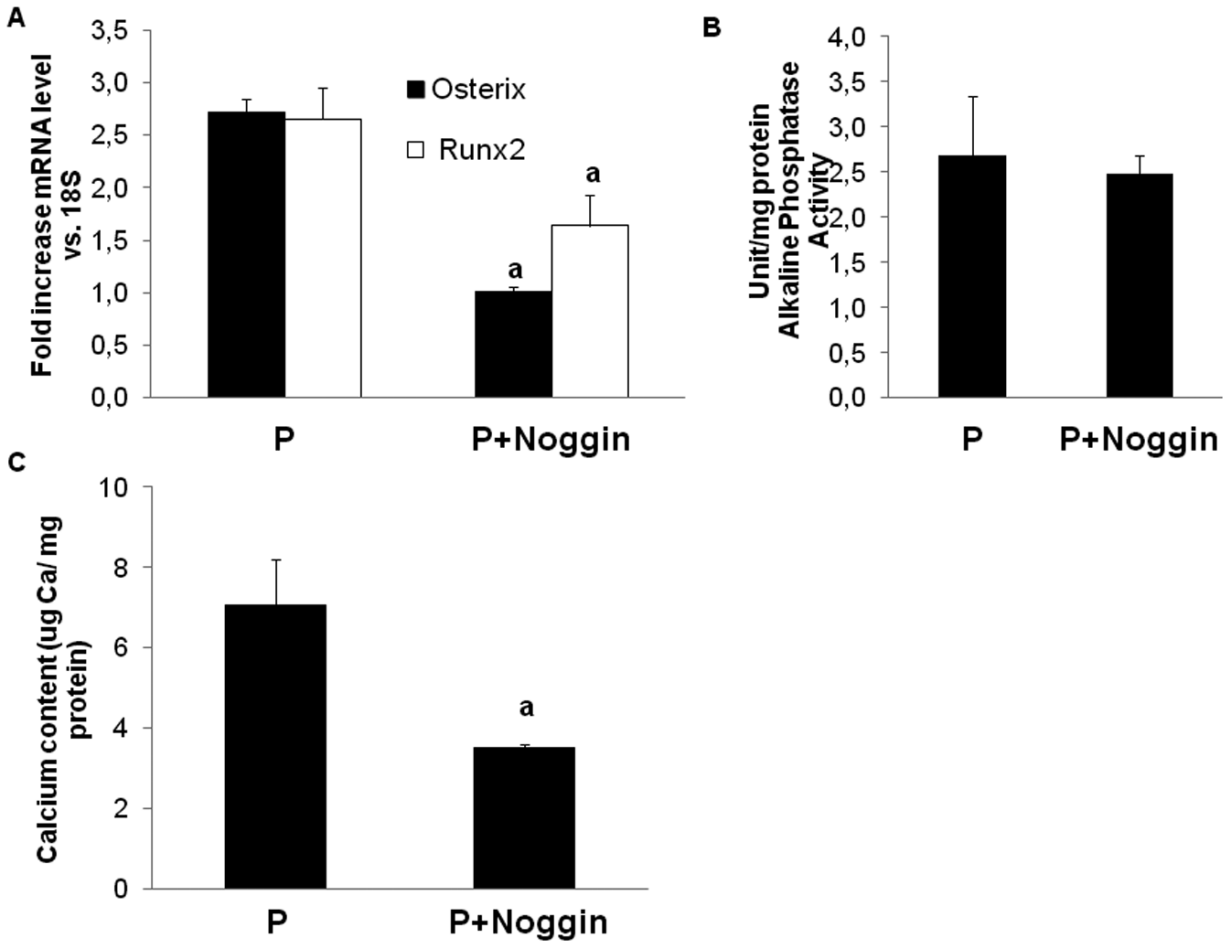
BMP2 inhibition prevents the osteogenic effects of high phosphate. A and B) Noggin administration (200 ng/ml) prevented the expression of osteogenic markers such as Osterix and Runx2 (a p<0.001 vs. high phosphate treated cells) and reduced calcium deposition. C) Alkaline phosphatase activity was not modified after Noggin administration. The figures are representative of at least three experiments.

One key downstream signal of BMP-2 activation is the nuclear translocation of Smad1/5/8 with the subsequent osteogenic cell differentiation and calcification. TGF-β inhibits nuclear translocation of Smad1/5/8 induced by high phosphate. Figure 3 shows that phospho-Smad 1/5/8 was not present in the nucleus of MSC cultured with TGF-β alone or with TGF-β plus high phosphate. However, cells treated with high phosphate alone showed nuclear translocation of phospho-Smad1/5/8, which was accompanied by a significant increase in phosphatase alkaline and calcium deposition. In these cells, the addition of Noggin, a well-characterized selective BMP inhibitor, was associated to a decrease in the expression of osteogenic genes Osx and Runx2 (Figure 4A) and a reduction in the cell culture calcium content (Figure 4B), while alkaline phosphatase activity was not modified (Figure 4C).

### TGF-β modulates Wnt/β-catenin activation induced by high phosphate

Our experiments have shown that TGF-β obstructs the increase in BMP-2 expression and Smad1/5/8 translocation induced by high phosphate. Activation of canonical Wnt/β-catenin signaling pathway is also involved in osteogenesis and it is associated with high levels of extracellular phosphate. Thus, we explored the effect of TGF-β plus high phosphate on Wnt/β-catenin activation. Beforehand we showed that high phosphate induces nuclear translocation of β-catenin in MSC with subsequent TCF/LEF promoter activation (Figure S2, Supporting information).

**Figure 5:**
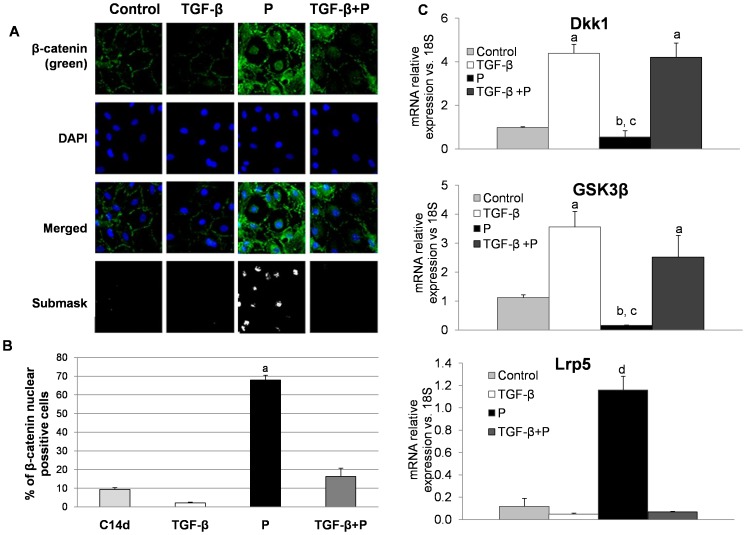
High phosphate activates Wnt/β-catenin pathway. A) Rat mesenchymal cells treated with TGF-β and/or high phosphate were stained for β-catenin immunofluorescence (green) and counterstained with DAPI (blue) to determine β-catenin subcellular localization. Merged images of β-catenin immunofluorescence and DAPI staining are shown. High phosphate induced nuclear translocation of β-catenin while the addition of TGF-β inhibited this translocation. Original magnification: 40x. Image is representative of three experiments. B) Quantification of β-catenin confocal immunofluorescence was performed with Image J software (a p<0.001 vs. all groups). C) With respect to control cells high phosphate decreased the expression of Dkk1 (b p<0.001) and Gsk3β (b p<0.001) while increased the expression of Lrp5 with respect to other groups (d p<0.001). These differences were also significant respect to TGF-β treated groups (c p<0.001 vs. TGF groups). TGF-β alone increased the expression of Dkk1 and Gsk3β (a p<0.001).

Confocal microscopy studies revealed that β-catenin was not observed in the nucleus of MSC treated with TGF-β alone. The addition of TGF-β to cells cultured with high phosphate avoided nuclear translocation of β-catenin induced by high phosphate alone (Figure 5A and B). Genes related to the activation of Wnt/β-catenin pathway were differentially modulated by TGF-β and high phosphate (Figure 5C). The expression of both Dkk1 and Gsk3β, which decrease Wnt/β-catenin activity, was reduced in cells exposed to high phosphate. By contrast, cells treated with TGF-β alone or TGF-β plus high phosphate showed increased expression of Dkk1and Gsk3β (Figure 5C). Conversely the expression of Lrp5, which activates Wnt/β-catenin, was high in cells treated with phosphate but it was reduced in cells treated with TGF-β alone or TGF-β plus high phosphate (Figure 5C).

### Wnt/β-catenin activity and BMP-2 expression in MSC

**Figure 6:**
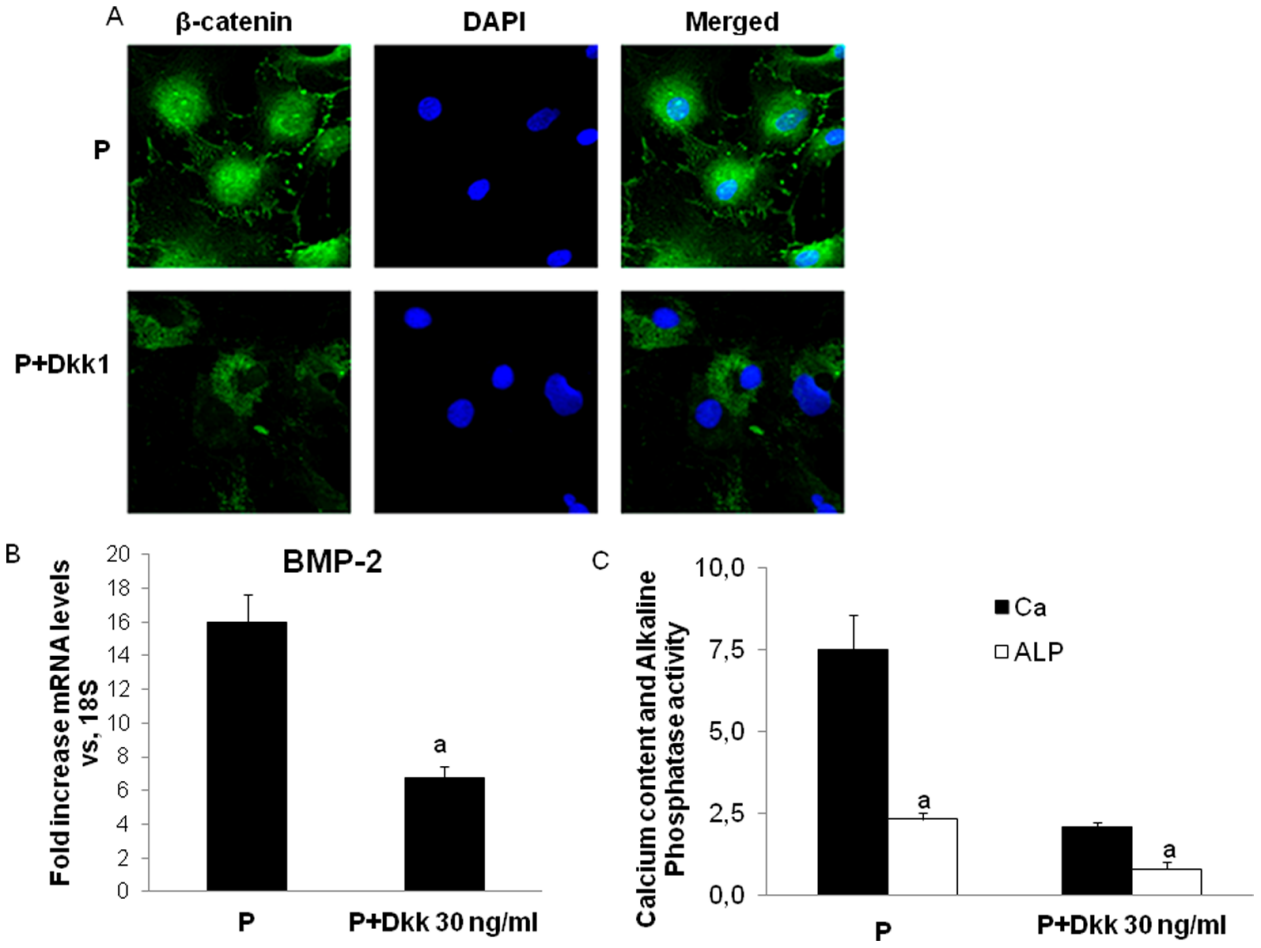
Dkk-1 inhibits the high phosphate-induced osteogenic-like characteristics in rat mesenchymal stem cells. A) Rat mesenchymal cells treated with high phosphate and Dkk-1 were stained for β-catenin immunofluorescence (green) and counterstained with DAPI (blue) to determine β-catenin subcellular localization. Merged images of β-catenin immunofluorescence and DAPI staining are shown. Dkk-1 administration reduced nuclear translocation of β-catenin. Original magnification: 40x. B) BMP-2 mRNA expression in rat mesenchymal stem cells treated with high phosphate and Dkk-1 was determined by RT-PCR (a p<0.001 vs high phosphate treated cells). C) Calcium content and alkaline phosphatase activity (Units/mg protein) in rat mesenchymal stem cells treated with high phosphate and Dkk-1 (a p<0.001 vs high phosphate alone). Image is representative of three experiments.

Inhibition of Wnt/β-catenin activity by Dkk-1 in MSC cultured with high phosphate (Figure 6A) was associated to a reduction of BMP-2 expression (Figure 6B) and a significant decrease in alkaline phosphatase activity and calcium deposition (Figure 6C).

**Figure 7:**
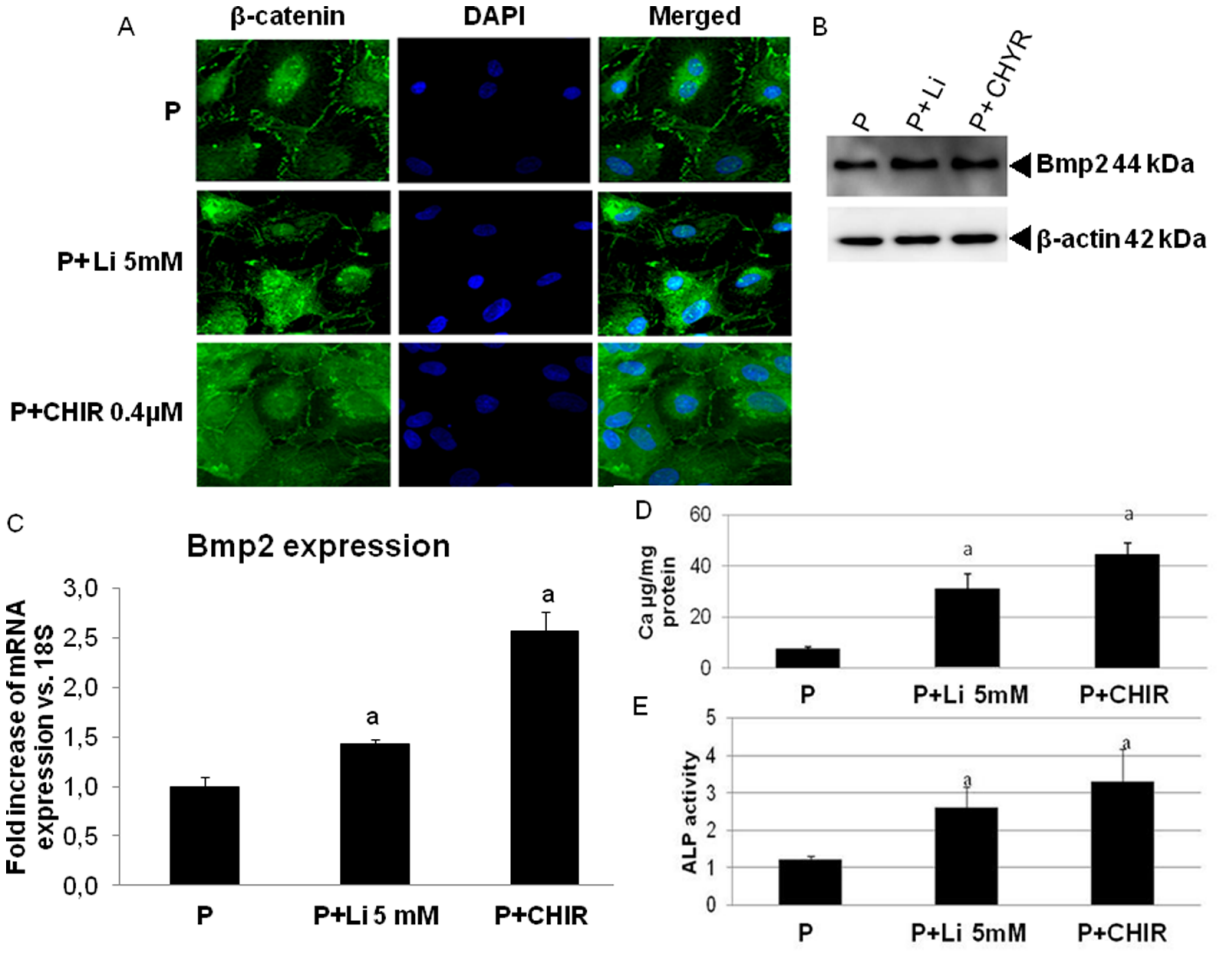
Wnt/β-catenin pathway activation enhances the high phosphate-induced osteogenic-like characteristics in rat mesenchymal stem cells. A) Rat mesenchymal cells treated with high phosphate and CHIR98014 (0.4 µM) or lithium chloride (5 mM) were stained for β-catenin immunofluorescence (green) and counterstained with DAPI (blue) to determine β-catenin subcellular localization. Merged images of β-catenin immunofluorescence and DAPI staining are shown. Both Wnt activators (CHIR98014 and lithium chloride) increased nuclear translocation of β-catenin. Original magnification: 40x. B) BMP-2 protein and C) mRNA expression in rat mesenchymal stem cells treated with high phosphate and CHIR98014 or lithium chloride was determined by western blot and RT-PCR respectively (a p < 0.001 vs high phosphate alone). D) Calcium content and E) alkaline phosphatase activity in rat mesenchymal stem cells treated with high phosphate and CHIR98014or lithium chloride (a p<0.001 vs. high phosphate alone). Image is representative of three experiments.

Thereafter, MSC cultured with high phosphate were treated with two different Wnt/β-catenin pathway activators, CHIR98014 and lithium chloride. As shown in figure 7A, both of them induced nuclear translocation of β-catenin, which was accompanied by an increase in BMP-2 expression (Figure 7B and C), alkaline phosphatase activity and calcium content (Figures 7D and E).

## Discussion

The main objective of this study was to evaluate in cultured MSC the signaling pathways involved in the regulation of the differentiation into VSMC and osteogenesis induced by TGF-β and high phosphate. The results demonstrated that TGF-β induces VSMC differentiation through nuclear translocation of Smad3. The addition of high phosphate prevents the VSMC differentiation induced by TGF-β. Nonetheless in the presence of TGF-β, high phosphate was unable to induce full osteogenic differentiation of MSC.

We have shown that TGF-β induces nuclear translocation of Smad 3. Similar results have been reported by other authors using human MSC [9]. In mature VSMC, TGF-β induces Smad3 binding to the SM22α promoter *in vivo* and myocardin cooperates with Smad3 to activate SM22α which enable these cells to maintain VSMC phenotype [11], [12].

A key question of this work was to determine whether high phosphate interferes with the TGF-β-induced VSMC differentiation. Our results show that the addition of high phosphate precludes VSMC differentiation induced by TGF-β; the expression of specific VSMC genes was inhibited by phosphate. Nuclear translocation of Smad3, which is essential for VSMC differentiation, was reduced by high phosphate (data not shown); therefore, it was not a surprise to observe a concomitant decrease in VSMC specific genes. These novel results illustrate the importance of nuclear translocation of Smad3 to achieve VSMC differentiation, revealing a mechanism whereby high phosphate interferes with normal VSMC differentiation.

In the absence of TGF-β, high phosphate induced the expression of the pro-osteogenic gene BMP-2; this was accompanied by an increase in alkaline phosphatase activity and calcification. These effects were mediated by nuclear translocation of Smad 1/5/8 and β-catenin and TGF- β prevented phosphate-induced nuclear translocation of these transcription factors essential for osteogenesis. While high phosphate prevented VSMC differentiation, the presence of TGF-β precluded phosphate-induced osteogenic differentiation of MSC. The pro-osteogenic role of BMP-2- Smad1/5/8 has been shown in human aortic valve interstitial cells from patients with calcifying aortic valve stenosis [13]-[14]. Our experiments show that high phosphate stimulates BMP-2 expression, which is accompanied by nuclear translocation of Smad 1/5/8. Noggin is a natural antagonist of bone morphogenetic proteins showing in MSC high affinity against BMP-2 and BMP-4 and less affinity for BMP-7 [15]. Noggin addition abrogated the osteogenic differentiation and the calcium deposition of high phosphate treated- MSC.

A similar effect was observed in cells incubated with high phosphate plus TGF-β. These results are in agreement with previous work [16] reporting that Noggin decreases the procalcific phenotype of human bone marrow-derived mesenchymal stem cells induced by uremic serum. Changes in BMP-2, BMP-4 or BMP-6 are detected during the phenotypic transition from VSMCs to osteoblasts-like cells [17]. Hayashi et al showed that addition of BMP2, BMP4 or BMP6 to cultured VSMC contributes to the loss of smooth muscle markers and the activation of Msx1 and Smad1/5/8 [18]. Osterix and Runx2 are downstream effectors of BMP signaling [19], [20]. Furthermore, the addition of calcitriol to human coronary artery smooth muscle cells enhanced osteogenesis and calcification of VSMC through an increase of BMP-6 [21]. However, other BMP such as BMP7 may in fact inhibit arterial calcification in the diabetic LDLR-/- mouse model [22].

Activation of Wnt/β-catenin pathway is also essential for osteogenic differentiation. Here we observed that high phosphate induces activation of Wnt/β-catenin pathway through nuclear translocation of β-catenin and activation of TCF/LEF promoter. It is well-known that high phosphate promotes osteogenic/chondrogenic differentiation of VSMC through Wnt/β-catenin activation [23], [24]. However, the effect of high phosphate on Wnt/β-catenin in MSC has not been explored in detail. In our study, the addition of the Wnt pathway inhibitor Dkk1, to MSC incubated in high phosphate prevented osteogenic differentiation. Similar effects were observed when TGF-β was added to MSC cultured in high phosphate medium: TGF-β prevented Wnt/β-catenin activation induced by high phosphate.

Other authors have also observed that high concentrations of TGF-β inhibit alkaline phosphatase activity and mineralization in MSC differentiated into osteoblasts [25], [26]. Ehnert et al have reported that the nuclear translocation of Smad 1/5/8 in osteoblasts was completely blocked by TGF- β via Smad7 up-regulation, Ski-related novel protein (SnoN) and Smurfs [27].

Regarding the inhibitory effects of TGF-β on Wnt signaling, it has been reported that TGF-β induces activation of noncanonical Wnt signaling with the subsequent inhibition of nuclear translocation of β-catenin [28], [29]. However, the results are not consistent and other authors have suggested that TGF-β might cooperate with β-catenin to induce osteoblastogenesis [30].

From our results, it seems clear that TGF-β decreases the activity of BMP-2 and canonical Wnt/β-catenin, two key pathways involved in osteogenesis of MSC. Nevertheless the interelationship between BMP-2 and canonical Wnt/β-catenin is not well defined [31]. Both are essential for osteogenic differentiation; our data show that the inhibition of BMP signaling by Noggin precluded the expression of osteogenic genes. We also observed that in presence of high phosphate, the inhibition of Wnt/β-catenin by Dkk1 administration is associated to a reduction of BMP-2 expression while the administration of Wnt activators resulted in an increase in BMP-2 expression. These results would suggest that canonical Wnt/β-catenin pathway is an upstream activator of BMP-2 expression in MSC as suggested by other authors [32], [33].

The interaction between TGF-β and high phosphate on the differentiation of MSC may have important implications in the repair of the damaged vascular wall. Regression of VC has been observed in experimental studies when the factor promoting calcification is removed [34]. Presumably, healing of the damaged tissue will require remodeling of the vascular wall with the incorporation of progenitor cells [35], [36]. Our results indicate that in the presence of high phosphate (e.g. in chronic kidney disease patients with hyperphosphatemia) these repairing cells will likely drift toward an osteogenic phenotype, instead of being committed to VSMC differentiation.

In conclusion, our results show a crosstalk between the effects triggered by TGF-β and high phosphate in cultured MSC. VSMC differentiation induced by TGF-β can not be achieved in the presence of high levels of phosphate. On the other hand, TGF-β obstructs phosphate induced osteogenesis by reducing the activity of Wnt/β-catenin and BMP pathways

## Supporting Information

Figure S1 **A.** Immunophenotype of rat MSC (mean ± stdesv). **B.** Osteogenic differentiation of rat mesenchymal stem cells. Alizarin Red Staining after 21 days of differentiation with Dexamethasone (1 uM), ascorbic acid (0.2 mM) and β-glicerolphosphate (10 mM). Image is representative of three experiments. **C.** Adypogenic differentiation of rat mesenchymal stem cells. Lipid drops were visible under the inverted microscope after 14 days of culture. Image is representative of three experiments.
(TIF)

Figure S2 **A.** Mesenchymal stem cells were nucleofected with pGL3-OT, pRL-CMV or pmax GFP and treated with high phosphate for 24h. The ratio of the luciferase activity from a TCF-responsive reporter construct (pGL3-OT) and a control luciferase reporter gene construct (pRL-CMV) representing Wnt/β-catenin pathway activation increased approximately 2.5 after high phosphate treatment compared to untreated control cells (a p<0.001 vs. Control cells). **B.** Green Fluorescent protein was nucleofected with Amaxa kit in order to check the efficiency of nucleofection.
(TIF)
