# Correction: TGF-β Prevents Phosphate-Induced Osteogenesis through Inhibition of BMP and Wnt/β-Catenin Pathways

**DOI:** 10.1371/journal.pone.0101910

**Published:** 2014-06-27

**Authors:** The PLOS ONE Staff

There are errors in the Funding section. The correct funding information is as follows: This study has been supported by ISCIII and Fondos FEDER (FIS PI11/02055), “Proyecto de Excelencia P09-CTS-5205” from Consejería de Economía, Ciencia y Empresa from Junta de Andalucía, PI-0127 from Consejería de Salud, Junta de Andalucia and UE Grant from Framework Programme 7 Syskid (FP7-241544). The funders had no role in study design, data collection and analysis, decision to publish, or preparation of the manuscript.
