# Supplementary figures and images for: TGF-β Prevents Phosphate-Induced Osteogenesis through Inhibition of BMP and Wnt/β-Catenin Pathways
Source: PLoS One. 2014 Feb 27;9(2):e89179. doi: 10.1371/journal.pone.0089179 (PMC3937350; doi:10.1371/journal.pone.0089179)

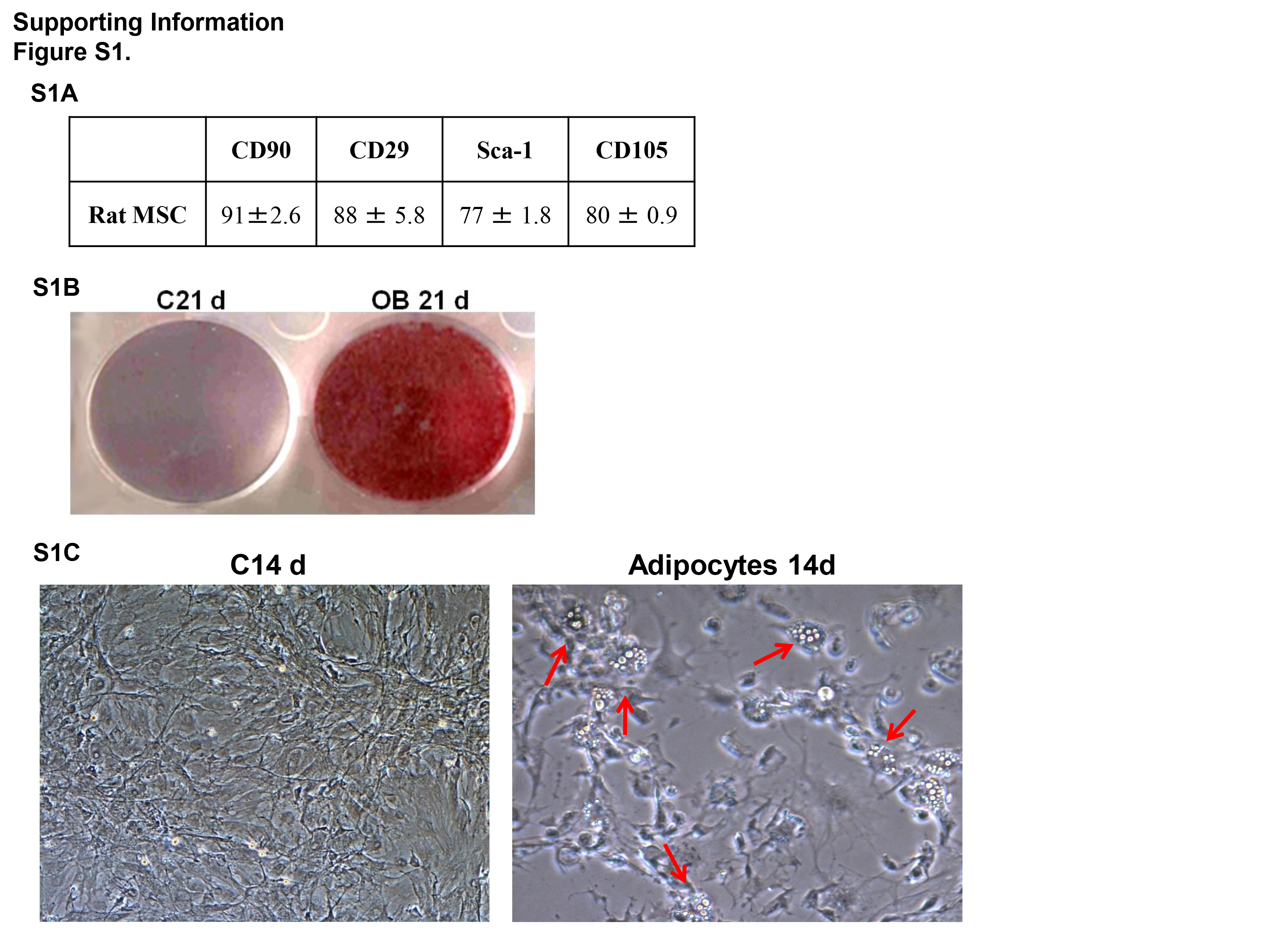

Supplement: Figure S1 — A. Immunophenotype of rat MSC (mean ± stdesv). B. Osteogenic differentiation of rat mesenchymal stem cells. Alizarin Red Staining after 21 days of differentiation with Dexamethasone (1 uM), ascorbic acid (0.2 mM) and β-glicerolphosphate (10 mM). Image is representative of three experiments. C. Adypogenic differentiation of rat mesenchymal stem cells. Lipid drops were visible under the inverted microscope after 14 days of culture. Image is representative of three experiments. (TIF) [file pone.0089179.s001.tif]

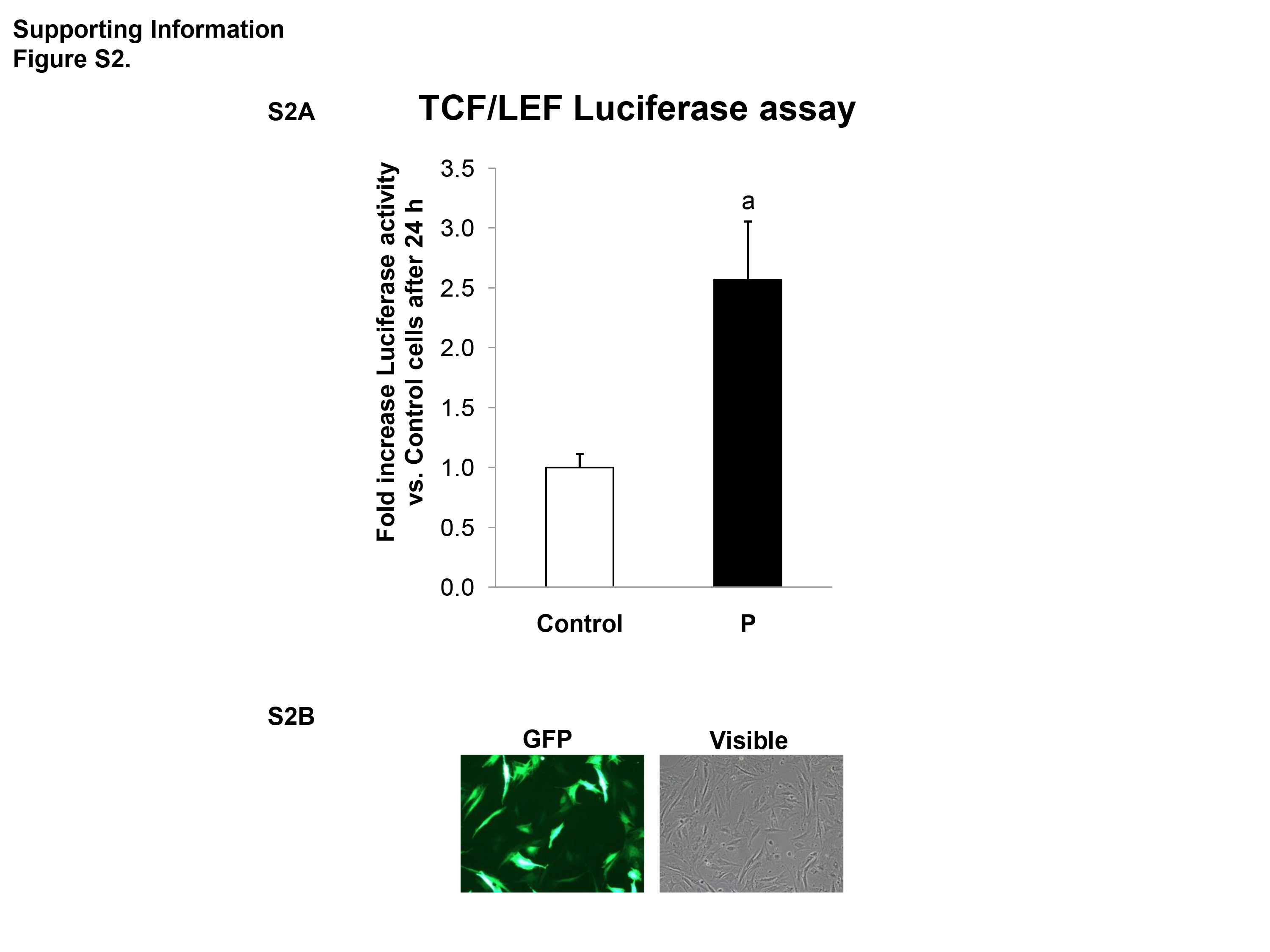

Supplement: Figure S2 — A. Mesenchymal stem cells were nucleofected with pGL3-OT, pRL-CMV or pmax GFP and treated with high phosphate for 24h. The ratio of the luciferase activity from a TCF-responsive reporter construct (pGL3-OT) and a control luciferase reporter gene construct (pRL-CMV) representing Wnt/β-catenin pathway activation increased approximately 2.5 after high phosphate treatment compared to untreated control cells (a p<0.001 vs. Control cells). B. Green Fluorescent protein was nucleofected with Amaxa kit in order to check the efficiency of nucleofection. (TIF) [file pone.0089179.s002.tif]
